# Multidimensional data analysis revealed thyroiditis-associated TCF19 SNP rs2073724 as a highly ranked protective variant in thyroid cancer

**DOI:** 10.18632/aging.205718

**Published:** 2024-04-04

**Authors:** Xianhui Ruan, Yu Liu, Shuping Wu, Guiming Fu, Mei Tao, Yue Huang, Dapeng Li, Songfeng Wei, Ming Gao, Shicheng Guo, Junya Ning, Xiangqian Zheng

**Affiliations:** 1 Department of Thyroid and Neck Tumor, Tianjin Medical University Cancer Institute and Hospital, National Clinical Research Center for Cancer, Tianjin’s Clinical Research Center for Cancer, Key Laboratory of Cancer Prevention and Therapy, Tianjin 300060, China; 2 Department of Head and Neck Surgery, Clinical Oncology School of Fujian Medical University, Fujian Cancer Hospital, Fuzhou 350014, Fujian, China; 3 Thyroid-Otolaryngology Department, Sichuan Clinical Research Center for Cancer, Sichuan Cancer Hospital and Institute, Sichuan Cancer Center, Affiliated Cancer Hospital of University of Electronic Science and Technology of China, Chengdu 610000, Sichuan, China; 4 Department of Thyroid and Breast Surgery, Tianjin Union Medical Center, Tianjin 300121, China; 5 Tianjin Key Laboratory of General Surgery in Construction, Tianjin Union Medical Center, Tianjin 300121, China; 6 Department of Medical Genetics, School of Medicine and Public Health, University of Wisconsin-Madison, Madison, WI 53706, USA

**Keywords:** thyroid cancer, GWAS, biomarker, therapeutic target, SNP

## Abstract

Background: Thyroid cancer represents the most prevalent malignant endocrine tumour, with rising incidence worldwide and high mortality rates among patients exhibiting dedifferentiation and metastasis. Effective biomarkers and therapeutic interventions are warranted in aggressive thyroid malignancies. The transcription factor 19 (TCF19) gene has been implicated in conferring a malignant phenotype in cancers. However, its contribution to thyroid neoplasms remains unclear.

Results: In this study, we performed genome-wide and phenome-wide association studies to identify a potential causal relationship between TCF19 and thyroid cancer. Our analyses revealed significant associations between TCF19 and various autoimmune diseases and human cancers, including cervical cancer and autoimmune thyroiditis, with a particularly robust signal for the deleterious missense variation rs2073724 that is associated with thyroid function, hypothyroidism, and autoimmunity. Furthermore, functional assays and transcriptional profiling in thyroid cancer cells demonstrated that TCF19 regulates important biological processes, especially inflammatory and immune responses. We demonstrated that TCF19 could promote the progression of thyroid cancer *in vitro* and *in vivo* and the C>T variant of rs2073724 disrupted TCF19 protein binding to target gene promoters and their expression, thus reversing the effect of TCF19 protein.

Conclusions: Taken together, these findings implicate TCF19 as a promising therapeutic target in aggressive thyroid malignancies and designate rs2073724 as a causal biomarker warranting further investigation in thyroid cancer.

## INTRODUCTION

Thyroid cancer is the most common malignant endocrine tumour, accounting for 1-2% of all malignant tumours [1]. Thyroid cancer is an important public health concern because its incidence has been increasing globally [2, 3]. A study conducted by the International Agency for Research on Cancer for GLOBOCAN 2020 and WHO cancer statistics estimated 224,023 and 53,815 new thyroid cancer cases in China and the United States in 2022, respectively, with 9,915 deaths (4.4%) and 2,262 deaths (4.2%) [4, 5]. Most thyroid cancers are indolent, but some tumours undergo dedifferentiation and metastasis, resulting in a high mortality rate [6–8]. These patients do not respond well to conventional treatments, including surgery and radioactive iodine (I^131^) [9, 10]. As a result, identifying effective biomarkers and therapeutic targets is crucial for aggressive thyroid cancer.

As a complex disease, thyroid cancer is related to multiple genetic factors. Recently, unbiased large-scale association studies have been conducted to identify genetic variations associated with an increased risk of developing thyroid cancer. For instance, a genome-wide association study (GWAS) identified 24 new genetic variants associated with thyroid stimulating hormone in 2020 [11]. Other studies have identified specific genes that may play a role in the development of thyroid cancer, including genes involved in DNA repair, cell cycle regulation, and immune function [12, 13]. In addition, a phenome-wide association study (PheWAS) was conducted to use electronic health record (EHR) data and identify genetic associations with multiple phenotypes, such as thyroid function, thyroid hormone levels, and thyroid nodules, simultaneously [14, 15]. Overall, these data-driven GWAS and PheWAS have provided valuable insights into the genetic basis of thyroid cancer and have the potential to lead to the development of new diagnostic and treatment strategies. However, further research is needed to fully understand the complex genetic and environmental factors that contribute to thyroid cancer development and progression. In addition, like all large-scale association studies, findings from both GWAS and PheWAS must be validated before they can be considered definitive.

In the current study, we comprehensively collected previous GWAS and PheWAS studies for thyroid-related diseases. By further leveraging large-scale genetic expression data, we prioritized a key gene, TCF19, which was demonstrated to have a potential causal relationship with thyroid cancer. Moreover, we performed *in vitro* functional studies and found that TCF19 could significantly promote the proliferation and metastasis of thyroid cancer and increase the severity of disease progression. An important finding was that the C>T variant of rs2073724 partially blocked TCF19’s function and played a protective role in thyroid cancer progression. RNA-seq analysis of TCF19 wild-type and SNP-overexpressing cells illustrated that TCF19 regulates many important biological processes in thyroid cancer, especially the inflammation pathway and the immune response [16].

## RESULTS

### Genome-wide and phenome-wide associations between TCF19 and immune diseases and human cancers

We collected multiple GWAS studies, including UKB, FinnGen, and GWAS catalogue, and identified that TCF19 is significantly associated with multiple autoimmune diseases and human cancers, for example, type 1 diabetes and autoimmune thyroid diseases (P=1.0x10^-23^), cervical cancer (4.0x10^-25^) and lung cancer (5.0x10^-19^) (Table 1). We also observed that rs2073724, a potential deleterious missense variation predicted by Sorting Intolerant From Tolerant (SIFT) [16], is associated with thyroid function (P=2.9x10-19), thyroiditis (P=2.3x10-7), hypothyroidism (P=1.0x10-8) and multiple autoimmune diseases (P=2.7x10^-10^) (Table 2).

**Table 1:** Previous PheWAS studies that are reported to be associated with TCF19 gene.

**Study ID**	**Reported trait**	**Study N cases**	**Index variant ID**	**Index variant RSID**	***p*-value**	**PMID**	**Gene**
GCST90011816	Cervical cancer	416913	6_31409942_T_C	rs2523496	4E-25	PMID:32887889	TCF19
GCST002876	Type 1 diabetes and autoimmune thyroid diseases	1598	6_31600692_G_A	rs2857595	2E-23	PMID:25936594	TCF19
GCST008365	Thyrotoxic hypokalemic periodic paralysis and Graves disease	3782	6_31090401_T_C	rs4947296	3E-22	PMID:31050781	TCF19
GCST90018770	Medication use (thyroid preparations)	178726	6_31593738_C_T	rs9357135	1E-19	PMID:34594039	TCF19
GCST012213	Aerodigestive squamous cell cancer (pleiotropy)	75848	6_31459618_G_C	rs9267123	2E-19	PMID:33667223	TCF19
GCST004748	Lung cancer	85716	6_31466334_A_G	rs3094604	5E-19	PMID:28604730	TCF19
GCST001900	Cervical cancer	7180	6_31422633_T_C	rs2516448	4E-18	PMID:23482656	TCF19
GCST012475	Cervical intraepithelial neoplasia grade 3 and invasive cervical cancer	150314	6_31410016_T_A	rs6938453	2E-17	PMID:33794208	TCF19
GCST012213	Aerodigestive squamous cell cancer (pleiotropy)	75848	6_30731245_G_A	rs3095327	2E-16	PMID:33667223	TCF19
GCST90018921	Skin cancer	670929	6_31356770_G_C	rs1065386	4E-16	PMID:34594039	TCF19
GCST006899	Thyroid stimulating hormone levels	76671	6_31140352_C_T	rs1265091	5E-15	PMID:30367059	TCF19
GCST90011822	Cancer (pleiotropy)	475312	6_31346329_C_G	rs3016018	2E-14	PMID:32887889	TCF19
GCST002876	Type 1 diabetes and autoimmune thyroid diseases	1598	6_30814225_C_T	rs886424	3E-14	PMID:25936594	TCF19
GCST004833	Cervical cancer	9347	6_31512891_G_A	rs3132461	2E-13	PMID:28806749	TCF19
GCST011008	Autoimmune traits	64039	6_31380039_G_A	rs4713462	2E-13	PMID:32534018	TCF19
GCST008870	Keratinocyte cancer (MTAG)	358840	6_31441506_G_A	rs61447909	4E-13	PMID:31174203	TCF19
GCST90011816	Cervical cancer	416913	6_31021915_T_G	rs114115115	6E-13	PMID:32887889	TCF19
GCST008573	Composite immunoglobulin trait (IgA/IgM)	16329	6_31358474_C_T	rs9405084	8E-13	PMID:28628107	TCF19
GCST90027058	Non-melanoma skin cancer	274830	6_31137533_A_G	rs1265100	8E-13	PMID:34290314	TCF19
GCST90085699	Human papilloma virus 16 positive oropharyngeal cancer	6334	6_31363011_G_A	rs9266329	1E-12	PMID:34642315	TCF19
GCST90018849	Gastric cancer	643238	6_31281955_T_C	rs2853940	8E-12	PMID:34594039	TCF19
GCST008870	Keratinocyte cancer (MTAG)	358840	6_31394488_T_C	rs28366119	4E-11	PMID:31174203	TCF19
GCST90018905	Prostate cancer	301559	6_31099487_C_T	rs9263523	1E-10	PMID:34594039	TCF19
GCST008870	Keratinocyte cancer (MTAG)	358840	6_31346869_A_G	rs2507999	1E-10	PMID:31174203	TCF19
GCST010286	Oropharynx cancer	11197	6_30991224_G_A	rs13211972	1E-10	PMID:32276964	TCF19
GCST90018629	Gastric cancer	167122	6_31271808_C_T	rs1050437	2E-10	PMID:34594039	TCF19
GCST90011823	Cancer (pleiotropy) (bidirectional)	475312	6_31045574_G_A	rs17190106	2E-10	PMID:32887889	TCF19
GCST011932	Thyrotoxic periodic paralysis	3822	6_31466032_T_G	rs112723370	5E-10	PMID:33105104	TCF19
GCST008500	Tetanus toxoid IgG concentrations post childhood immunization	1852	6_31481245_T_C	rs2523650	1E-09	PMID:31189108	TCF19
GCST003527_4	Anti-thyroid drug induced agranulocytosis	5209	6_31356759_T_A	rs1071816	2E-09	PMID:27157822	TCF19
GCST009825	Cervical cancer (MTAG)	40094	6_30719695_C_T	rs117670375	2E-09	PMID:31488892	TCF19
GCST90085699	Human papilloma virus 16 positive oropharyngeal cancer	6334	6_31466557_C_T	rs2523679	3E-09	PMID:34642315	TCF19
GCST009823_8	Gynecologic disease	47331	6_31404941_G_A	rs2507968	3E-09	PMID:31488892	TCF19
GCST007856	Colorectal cancer or advanced adenoma	125478	6_31042408_T_C	rs116353863	9E-09	PMID:30510241	TCF19
GCST001474	Hypothyroidism	39282	6_31050630_A_G	rs2517532	1E-08	PMID:22493691	TCF19
GCST009823_5	Gynecologic disease (multivariate analysis) [uterine cervical cancer]	47331	6_30719695_C_T	rs117670375	1E-08	PMID:31488892	TCF19
GCST90018638	Hepatic cancer	161323	6_31355203_C_T	rs1131500	2E-08	PMID:34594039	TCF19
GCST007992	Colorectal cancer	92967	6_30790689_A_G	rs3131043	3E-08	PMID:31089142	TCF19
GCST001148	Prostate cancer	73609	6_31150734_T_G	rs130067	3E-08	PMID:21743467	TCF19
GCST009825	Cervical cancer (MTAG)	40094	6_31330644_T_C	rs28752857	5E-08	PMID:31488892	TCF19

Note: previously GWAS and pheWAS studies are collected from multiple sources including GWAS catalog, Opentargets and ExPheWas.

**Table 2:** Previous PheWAS studies that are reported to be associated with rs2073724 in TCF19.

**Study.ID**	**Trait**	***p*-value**	**N.Overall**	**SNP**	**Gene**	**PMID**
GCST90038635	Thyroid problem (not cancer)	2.9E-19	484598	rs2073724	TCF19	PMID:33959723
NEALE2_20002_1111	Asthma | non-cancer illness code, self-reported	6.84289E-19	361141	rs2073724	TCF19	UKB Neale v2
NEALE2_20002_1456	Malabsorption/coeliac disease | non-cancer illness code, self-reported	1.32417E-18	361141	rs2073724	TCF19	UKB Neale v2
NEALE2_20002_1226	Hypothyroidism/myxoedema | non-cancer illness code, self-reported	8.05449E-14	361141	rs2073724	TCF19	UKB Neale v2
FINNGEN_R6_D3_immuNEMECHANISM	Certain disorders involving the immune mechanism	1.35E-10	260405	rs2073724	TCF19	FINNGEN_R6
FINNGEN_R6_D3_BLOOD	Diseases of the blood and blood-forming organs and certain disorders involving the immune mechanism	1.82E-10	260405	rs2073724	TCF19	FINNGEN_R6
FINNGEN_R6_AUTOimmuNE	Autoimmune diseases	2.7E-10	260405	rs2073724	TCF19	FINNGEN_R6
FINNGEN_R6_CD2_FOLLICULAR_LYMPHOMA_EXALLC	Follicular lymphoma (controls excluding all cancers)	2.23E-09	209748	rs2073724	TCF19	FINNGEN_R6
GCST90038603	immunological or systemic disorder	0.000000029	484598	rs2073724	TCF19	PMID:33959723
NEALE2_20002_1428	Thyroiditis | non-cancer illness code, self-reported	2.32757E-07	361141	rs2073724	TCF19	UKB Neale v2
FINNGEN_R6_C3_UNCERTAIN_SECONDARY_EXALLC	Secondary uncertain malignant neoplasm (controls excluding all cancers)	0.00000067	206307	rs2073724	TCF19	FINNGEN_R6
FINNGEN_R6_C3_LYMPHNODES_SECONDARY_NAS_EXALLC	Secondary and unspecified malignant neoplasm of lymph nodes (controls excluding all cancers)	0.0000101	205451	rs2073724	TCF19	FINNGEN_R6
NEALE2_20002_1371	Sarcoidosis | non-cancer illness code, self-reported	2.48599E-05	361141	rs2073724	TCF19	UKB Neale v2
NEALE2_20002_1453	Psoriasis | non-cancer illness code, self-reported	5.71729E-05	361141	rs2073724	TCF19	UKB Neale v2
NEALE2_20002_1313	Ankylosing spondylitis | non-cancer illness code, self-reported	9.10283E-05	361141	rs2073724	TCF19	UKB Neale v2
FINNGEN_R6_CD2_INSITU_CERVIX_UTERI_EXALLC	Carcinoma *in situ* of cervix uteri (controls excluding all cancers)	0.00012	119317	rs2073724	TCF19	FINNGEN_R6
NEALE2_20002_1387	Hayfever/allergic rhinitis | non-cancer illness code, self-reported	0.000127793	361141	rs2073724	TCF19	UKB Neale v2
NEALE2_20002_1471	Atrial fibrillation | non-cancer illness code, self-reported	0.000132399	361141	rs2073724	TCF19	UKB Neale v2
GCST006085	Prostate cancer	0.000243	140254	rs2073724	TCF19	PMID:29892016
NEALE2_20002_1225	Hyperthyroidism/thyrotoxicosis | non-cancer illness code, self-reported	0.000245311	361141	rs2073724	TCF19	UKB Neale v2
FINNGEN_R6_C3_VULVA_EXALLC	Malignant neoplasm of vulva (controls excluding all cancers)	0.000323	117169	rs2073724	TCF19	FINNGEN_R6
NEALE2_20002_1473	High cholesterol | non-cancer illness code, self-reported	0.000333684	361141	rs2073724	TCF19	UKB Neale v2
FINNGEN_R6_C3_CERVIX_UTERI_EXALLC	Malignant neoplasm of cervix uteri (controls excluding all cancers)	0.000981	119210	rs2073724	TCF19	FINNGEN_R6
NEALE2_20002_1138	Gastro-oesophageal reflux (gord) / gastric reflux | non-cancer illness code, self-reported	0.00153374	361141	rs2073724	TCF19	UKB Neale v2
GCST006465	Endometrial cancer (endometrioid histology)	0.00253884	54884	rs2073724	TCF19	PMID:30093612
NEALE2_20002_1202	Urinary frequency / incontinence | non-cancer illness code, self-reported	0.00268392	361141	rs2073724	TCF19	UKB Neale v2
FINNGEN_R6_C3_DLBCL_EXALLC	Diffuse large B-cell lymphoma (controls excluding all cancers)	0.00283	204397	rs2073724	TCF19	FINNGEN_R6
NEALE2_20002_1597	Tinnitus / tiniitis | non-cancer illness code, self-reported	0.0031828	361141	rs2073724	TCF19	UKB Neale v2
GCST006464	Endometrial cancer	0.0040379	121885	rs2073724	TCF19	PMID:30093612
FINNGEN_R6_CD2_NONHODGKIN_NAS_EXALLC	Other and unspecified types of non-Hodgkin lymphoma (controls excluding all cancers)	0.0041	209777	rs2073724	TCF19	FINNGEN_R6
NEALE2_20002_1575	Herpes simplex | non-cancer illness code, self-reported	0.00427488	361141	rs2073724	TCF19	UKB Neale v2
SAIGE_149	Cancer of larynx, pharynx, nasal cavities	0.00458	407449	rs2073724	TCF19	UKB SAIGE
NEALE2_20002_1477	Psoriatic arthropathy | non-cancer illness code, self-reported	0.00466974	361141	rs2073724	TCF19	UKB Neale v2

Note: previously GWAS and pheWAS studies are collected from multiple sources including GWAS catalog, Opentargets and ExPheWas.

Moreover, the GTEx dataset showed that compared with the wild type allele (Pro), the rs2073724 alternative allele (Leu) increased the expression level of TCF19 in almost all human tissues (thyroid tissue, NES=0.99, P=9.6x10^-66^, Supplementary Figure 1). The causal relationship between the alternative allele and increased aetiology of thyroiditis and thyroid cancer. Through analysis of the TCF19-related SNP in UCSC, we found that rs2073724 was located in exon 3 of TCF19 (Supplementary Figure 2). Hence, exonic SNPs are important SNPs, and the function of exonic SNPs in thyroid cancer remains largely unknown. Therefore, the potential role of TCF19 and its exonic SNP (rs2073724) in thyroid cancer tumorigenesis and the specific mechanism drew our attention.

### TCF19 is upregulated in thyroid cancer and correlated with cancer progression and a poor prognosis

**Figure 1:**
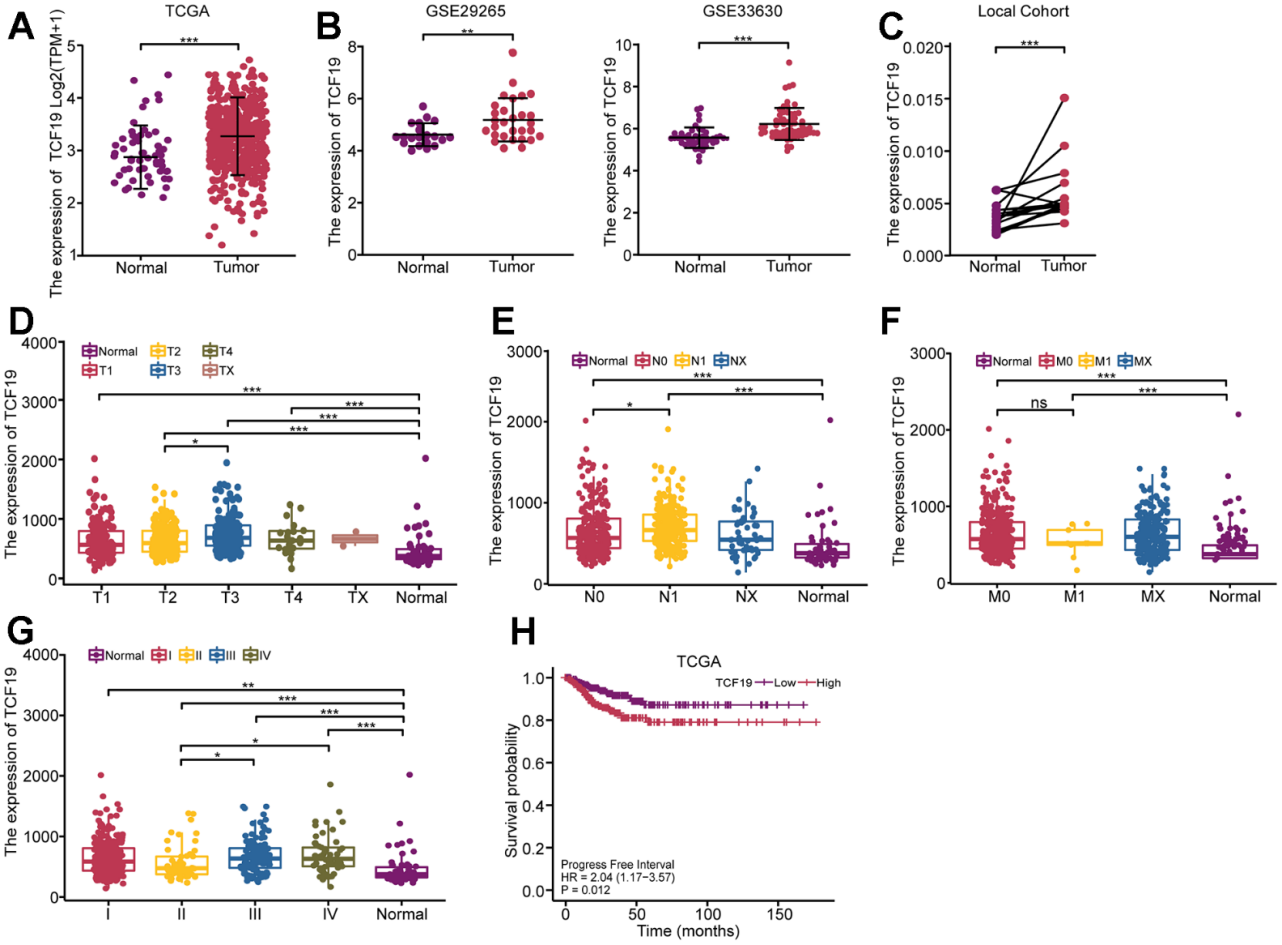
**High expression of TCF19 is associated with poor prognosis in thyroid cancer.** (**A**) The expression pattern of TCF19 in total was analyzed in 512 THCA tissues and 59 normal controls (TCGA database). (**B**) Kaplan-Meier analysis evaluating the association between Progress Free Interval and TCF19 expression in thyroid cancer patients (N=512) in the TCGA-THCA cohort. Progress Free Interval analysis was further stratified by TCF19-High and TCF19-Low characteristics for Kaplan–Meier analysis. (**C**) The expression pattern of TCF19 in GSE29265 and GSE33630, comparing normal thyroid tissues with thyroid cancer. (**D**–**G**) The relationship between TCF19 expression and T stage (**D**), N stage (**E**), M stage (**F**) and TNM stage (**G**) in TCGA cohort. (**H**) The expression pattern of TCF19 in 20 THCA tumor and their adjacent tissues. *p<0.05, **p < 0.01, ***p<0.001, NS, not significant.

To further investigate the expression pattern of TCF19 in human cancers, we first analysed The Cancer Genome Atlas (TCGA) database and found that TCF19 mRNA levels were upregulated in most human cancers (meta-analysis, log2FC=0.68, P=2.84x10^-13^) including thyroid cancer (Supplementary Figure 3 and Figure 1A).

Furthermore, in two independent GEO datasets (GSE29265 and GSE33630), TCF19 mRNA levels were significantly higher in thyroid cancer than in matched normal controls (Figure 1B). To confirm the results of the datasets, we used a quantitative reverse transcription PCR (RT-PCR) method to quantify TCF19 mRNA levels in 10 pairs of thyroid cancer tissues and their corresponding adjacent normal thyroid tissues. Upregulation of TCF19 was detected in 10 pairs, accounting for 90% of all tested samples (Figure 1C). We also analyzed the TCF19 expression levels in relation to TNM stages in thyroid cancer patients using the TCGA database. We observed that TCF19 expression was elevated in T3 stage primary lesions as compared to T2 (Figure 1D). Similarly, patients with lymph node metastasis exhibited higher TCF19 levels than those without (Figure 1E). Although no significant differences were found in metastasis (M), this could be due to the small number of cases with distant metastasis (M1) in our sample (Figure 1F). Moreover, TCF19 expression was significantly higher in stage III and IV tumors compared to stage II (Figure 1G). Furthermore, patients with high TCF19 expression had shorter progression-free intervals (PFIs) than those with low TCF19 expression (HR=2.04, p=0.012) (Figure 1H). These results indicate that TCF19 is highly expressed in thyroid cancer and that patients with high TCF19 expression have a poor prognosis.

### TCF19 promotes thyroid cancer progression, and the C>T variant of rs2073724 plays a protective role in thyroid cancer tumorigenesis

**Figure 2:**
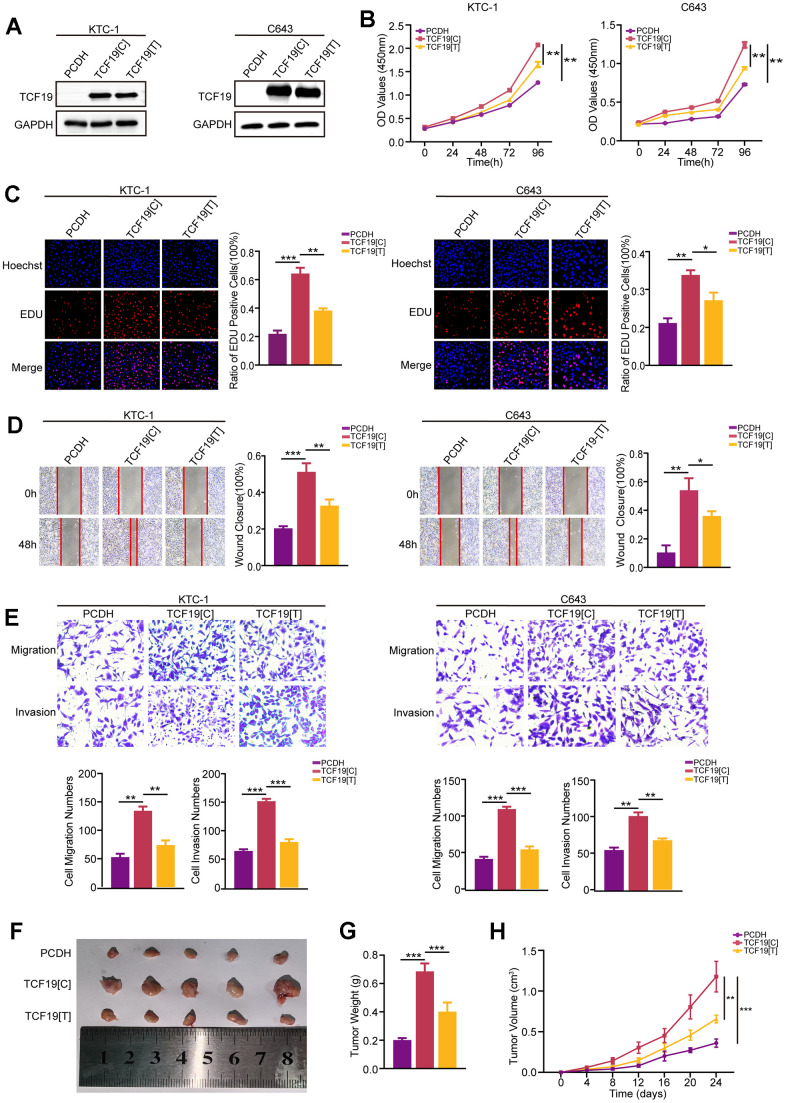
**TCF19[C] and TCF19[T] expression impacts thyroid cancer progression.** (**A**) TCF19 expression was detected in KTC-1 and C643 cells transfected with pcDNA, TCF19[C] and TCF19[T] plasmids by Western blotting. (**B**) CCK8 assay was used to detect cell viability. (**C**) The EdU method was used to detect DNA synthesis. (**D**) Wound healing assay was used to detect cell migration. (**E**) Transwell assay was used to detect cell invasion and migration in the cells indicated above. (**F**) Tumor images in tumor-bearing mouse groups. (**G**) Mouse tumor weights in various groups. (**H**) Mouse tumor volumes were measured every four days. Data are shown as means ± S.D. *p < 0.05, **p < 0.01, ***p < 0.001, NS, not significant.

To detect the function of TCF19 and the SNP in thyroid cancer progression, we established TCF19[C] (rs2073724[C]) expression and TCF19[T] (rs2073724[T]) overexpression in thyroid cancer. RT-qPCR and Western blot analysis confirmed that TCF19 was strongly overexpressed. There was no significant difference in the expression level between TCF19[C] and TCF19[T] (Figure 2A and Supplementary Figure 4A). CCK-8 and EdU assays revealed that TCF19[C] overexpression promotes thyroid cancer cell proliferation, and TCF19[T] partly blocked these effects (Figure 2B, 2C). Additionally, we also performed Transwell and wound healing assays. As we observed previously TCF19[C] significantly increased the migration and invasion of thyroid cancer cells, while overexpression of TCF19[T] notably reduced these abilities affected by TCF19[C] overexpression (Figure 2D). Taken together, TCF19 plays an oncogenic role in thyroid cancer progression. Notably, the alternative allele of the SNP is required for the effect of TCF19 on thyroid cancer progression. In addition, we also established stable TCF19-knockdown TC cells (Supplementary Figure 4A, 4B). As expected, downregulation of TCF19 suppressed the proliferation, migration, and invasion of thyroid cancer cells compared with control cells, as demonstrated by CCK-8, EdU, Transwell, and wound healing assays (Supplementary Figure 4C–4F). These results indicate that TCF19 regulates the proliferation, migration and invasion of thyroid cancer cells *in vitro*. To investigate TCF19’s function in tumorigenesis *in vivo*, we conducted subcutaneous tumor formation assays using nude mice. We injected nude mice subcutaneously with C643 cells overexpressing either TCF19[C] or TCF19[T], along with a control group. The mice were euthanized 24 days post-injection to assess tumor development. Our findings revealed that TCF19[C] overexpression facilitated the growth of subcutaneous tumors. In contrast, TCF19[T] overexpression was observed to impede tumor progression when compared to TCF19[C] (Figure 2F–2H). More importantly, the C>T variant of rs2073724 diminishes the carcinogenic effect of TCF19.

### Identification of TCF19[C] and TCF19[T] targets by RNA-seq

**Figure 3:**
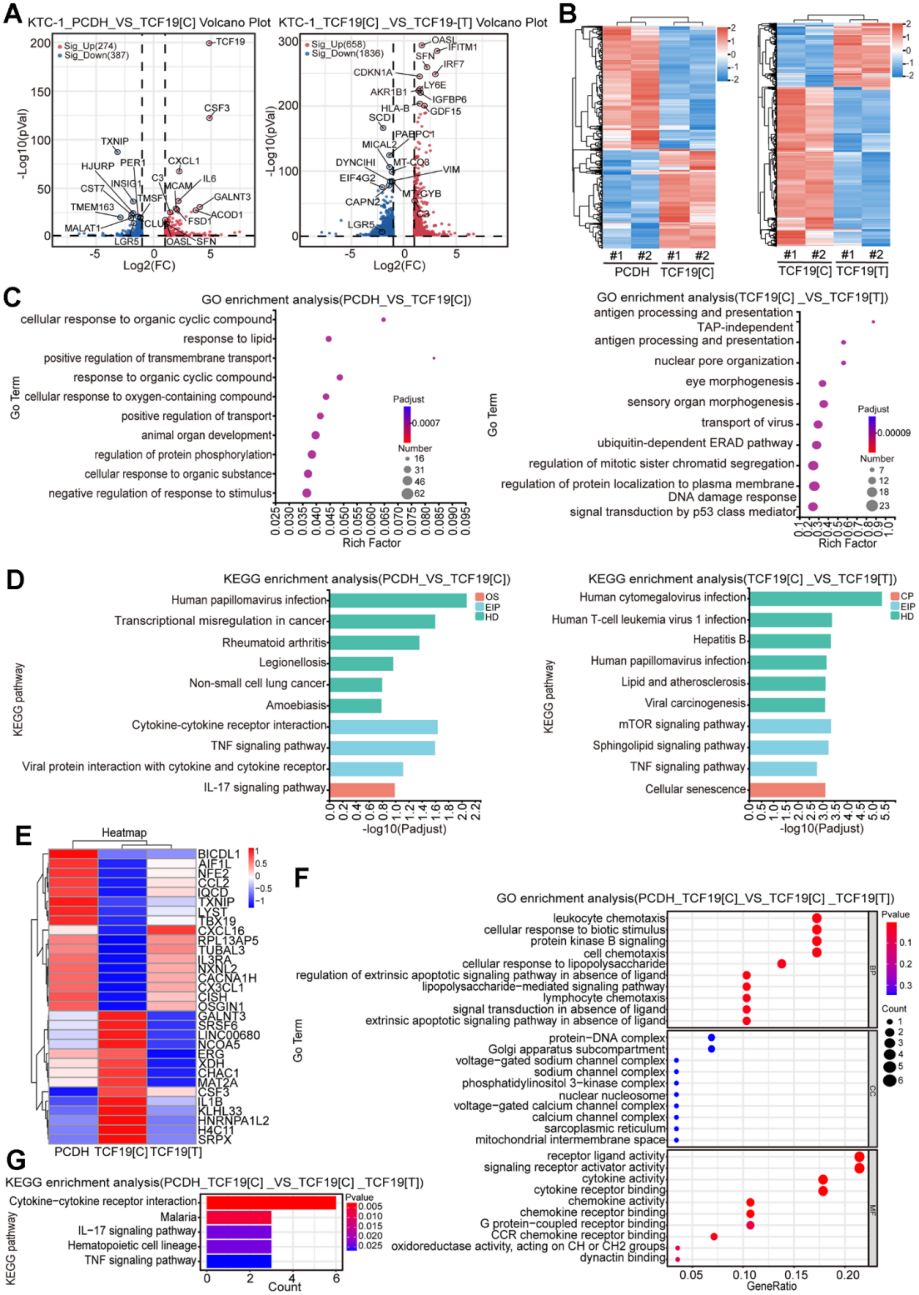
**Identification of TCF19[C] and TCF19[T] targets by RNA-seq.** (**A**) Volcano plots displaying differentially expressed genes (DEGs) in microarray data comparing PCDH with TCF19[C] and TCF19[C] with TCF19[T] KTC-1 cells. The numbers of significantly variant genes (FC > 2.0, P < 0.05) were shown. Vertical dashed lines indicate cut-off of FC (2.0), whereas the horizontal dashed lines indicate cut-off of *p*-value (0.05). (**B**) Heatmap of DEGs identified by RNA-seq. (**C**) Gene Ontology (GO) enrichment analysis of DEGs, *p*-value<0.05. (**D**) Kyoto Encyclopedia of Genes and Genomes (KEGG) enrichment analysis of DEGs, *p*-value<0.05. (**E**) Heatmap analyzed from 31 total genes obtained by the intersection of cluster 1 (PCDH vs TCF19[C]) and cluster 2 (TCF19[C] VS TCF19[T]). (**F**) GO analysis of genes described in (**E**). (**G**) KEGG analysis of genes described in (**E**).

To identify the potential targets regulated by TCF19, RNA sequencing (RNA-seq) was performed in control, TCF19[C] and TCF19[T] overexpression cells. As shown in Supplementary Figure 5A, PCA analysis indicates that the different RNA-seq samples could be completely distinguished. Overexpression of TCF19[C] resulted in abnormal gene expression, with 339 upregulated genes and 424 downregulated genes. However, when TCF19[T] was overexpressed, a total of 723 genes were upregulated and 1865 genes were downregulated in these cells compared to TCF19[C]-overexpressing cells (Figure 3A, 3B). The results indicate that TCF19[C] and TCF19[T] affect the expression of many genes in thyroid cancer cells. GO and KEGG analyses showed that the differentially expressed genes (DEGs) were mainly enriched in cancer-related programs or signalling pathways, such as the TNF signalling pathway, IL-17 signalling pathway and cytokine-cytokine receptor interaction (Figure 3C, 3D). And then we found that there were 31 genes associated with SNP (Figure 3E and Supplementary Figure 5B). Additionally, GO and KEGG analyses revealed enrichment of signatures associated with signalling receptor activator activity, the IL-17 signalling pathway, and the TNF signalling pathway (Figure 3F, 3G). Based on these results, TCF19 appears to be a potent regulator of thyroid cancer progression, especially in regard to the inflammatory response. In addition, the SNP rs2073724 plays a crucial role in maintaining protein function.

The tumour microenvironment (TME) is a complex and evolving system. Tumour occurrence, growth and metastasis strongly depend on the internal environment of tumour cells. It is related not only to the structure, function and metabolism of tumour tissues but also to the internal environment of tumour cells [17, 18]. To further stress the role of TCF19 in regulating the TME of thyroid cancer. We analysed the TCGA databases and found a positive correlation between TCF19 and immune scores (Supplementary Figure 6A) and stromal scores (Supplementary Figure 6B) and a negative correlation between TCF19 and tumour purity (Supplementary Figure 6C) in thyroid cancer. We further performed xCell and EPIC analyses to explore whether TCF19 expression correlates with immune cell infiltration. The results showed that THCA samples with high TCF19 expression had higher levels of monocyte and macrophage infiltration than samples with low TCF19 expression (Supplementary Figure 6D, 6E). Next, we quantified 8 immune cell populations and 2 nonimmune stromal populations (endothelial cells and fibroblasts) by MCP-counter scoring. The results showed that the proportions of B lineage, monocytic lineage, CD8+ T cells, cytotoxic lymphocytes, fibroblasts and myeloid dendritic cells were higher, while the proportion of endothelial cells was lower in the TCF19 high group than in the TCF19 low group (Supplementary Figure 6F). We also used the Tumour Immune Dysfunction and Exclusion (TIDE) algorithm [19] to predict the response to immune checkpoint blockade therapy (ICB). In general, high TIDE score was associated with a lower ICB response rate. Our results suggest that TCF19 overexpression may confer resistance to ICB therapy (Supplementary Figure 6G).

### TCF19[T] reduces its DNA binding ability, thus affecting protein function

**Figure 4:**
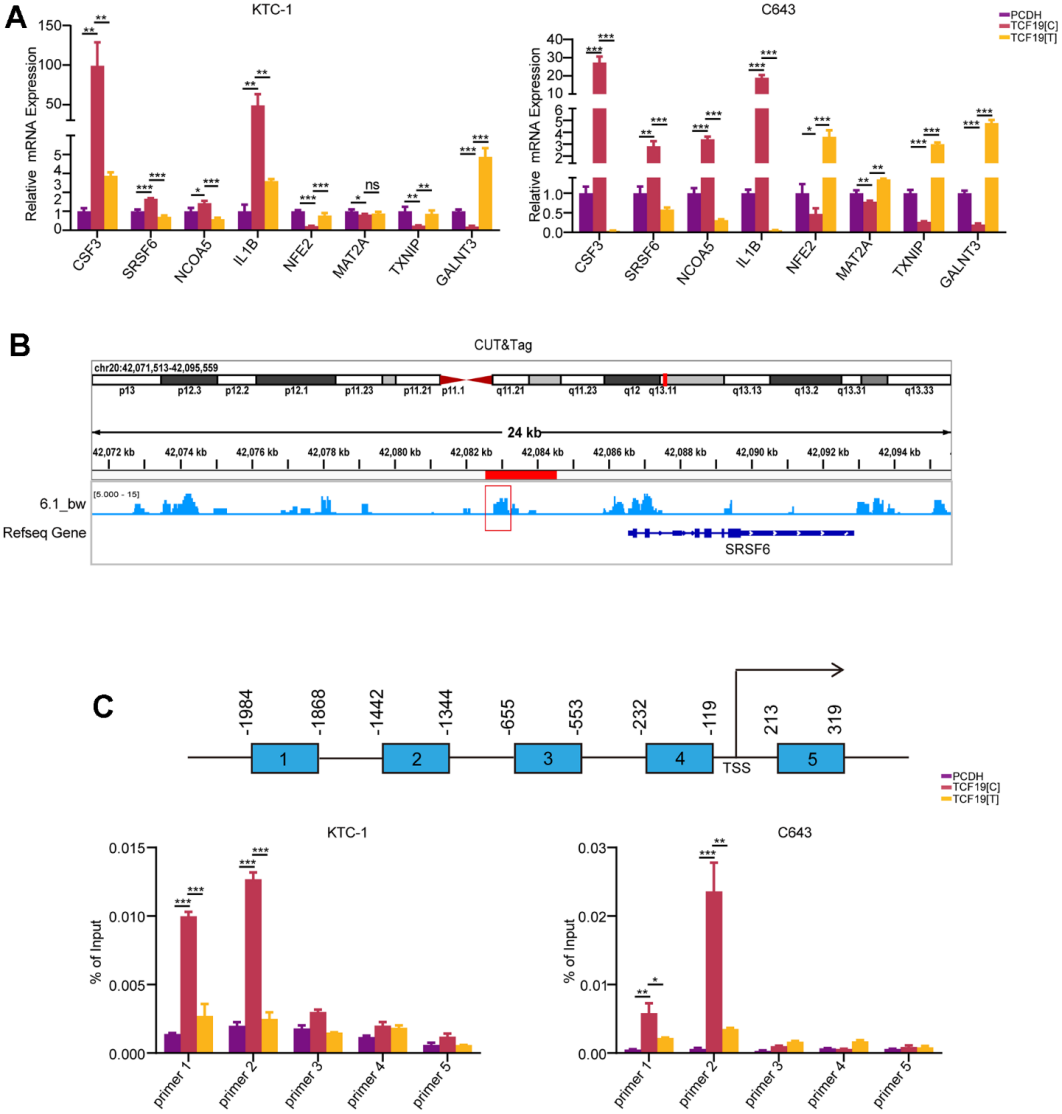
**TCF19[T] reduces its DNA binding ability, thus affecting protein function.** (**A**) CSF3, SRSF6, NCOA5, IL1B, NFE2, MAT2A, TXNIP, GALNT3 mRNA expression levels in cells stably transfected with PCDH, TCF19[C] and TCF19[T] plasmids were measured by qRT-PCR, respectively. (**B**) CUT&Tag data showed TCF19 could directly bind to the promoter of SRSF6 gene. (**C**) ChIP-qPCR analysis revealed potential TCF19-binding sites in the SRSF6 promoter region. After SNP mutation, the binding ability was weakened.

To further validate that the SNP mutation could abolish TCF19 protein function, we performed RT-PCR to detect the downstream genes of TCF19 from the RNA-seq. The results indicated that TCF19 could significantly regulate its target genes, while TCF19[T] partly abolished this function (Figure 4A). To further stress this interesting question, we analysed the SNP mutation site of the protein. We found that the SNP mutation site was located in the proline-rich region of TCF19 (Supplementary Figure 7A, 7B). A previous study indicated that TCF19 contains a proline-rich region, which is a common characteristic of transactivating factors [20]. We noticed that the SNP was located in the proline-rich region of the TCF19 protein. Therefore, we hypothesized that the SNP mutation affects the transactivating ability of TCF19. Then, we analysed our unpublished CUT&Tag data for TCF19 and found that TCF19 could directly bind to the promoter of the SRSF6 gene (Figure 4B). To further verify whether TCF19 activates the SRSF6 promoter through direct binding, we performed chromatin immunoprecipitation (ChIP) assays. As expected, TCF19 was recruited to the promoter sites. However, TCF19[T] strongly reduced the binding ability at the promoter of SRSF6 (Figure 4C). Taken together, TCF19 may be an exciting new therapeutic target for aggressive thyroid cancer. The alternative allele of the rs2073724 variant disrupts the oncogenic functions of TCF19, implicating the rs2073724 C>T variant as a causal genetic variant influencing thyroid cancer risk.

## DISCUSSION

In this study, we first conducted a large-scale data integration of genome-wide association studies (GWAS) and phenome-wide association studies (pheWAS) to identify potential genetic risk factors for overall survival in cancer patients. The association results further overlapped with gene expression data from GTEx, which contains DNA and RNA data from samples acquired from donors postmortem across various tissue sites, and data from the TCGA project, which contains cancer samples. The gene expression level was log2 transformed, and both fixed effect and random effect models were used for the aggregation. TCF19 and a potential deleterious missense variation (rs2073724) were identified to be associated with multiple autoimmune diseases and human cancers. Furthermore, we constructed TCF19[C] and TCF19[T] overexpression constructs and performed shRNA knockdown experiments to investigate the role of TCF19 in thyroid carcinoma cells. Additionally, we conducted RNA-seq to investigate the mechanisms of TCF19 in thyroid cancer progression.

Our meta-analysis of GWAS and pheWAS data identified an association between TCF19 expression levels and overall survival time in thyroid cancer patients. Specifically, high TCF19 expression levels were associated with a decreased overall survival time. These results suggest that TCF19 may be a potential prognostic biomarker for thyroid cancer. Moreover, *in vitro* and *vivo* functional studies highlighted that TCF19 plays an oncogenic role in thyroid cancer development, and RNA-seq indicated that TCF19 regulates many important biological processes, especially inflammation pathways. Interestingly, the SNP (rs2073724) located at TCF19 partly abolished its role in cancer progression, and GWAS data showed that the C>T variant of rs2073724 is a deleterious missense variation.

**Figure 5:**
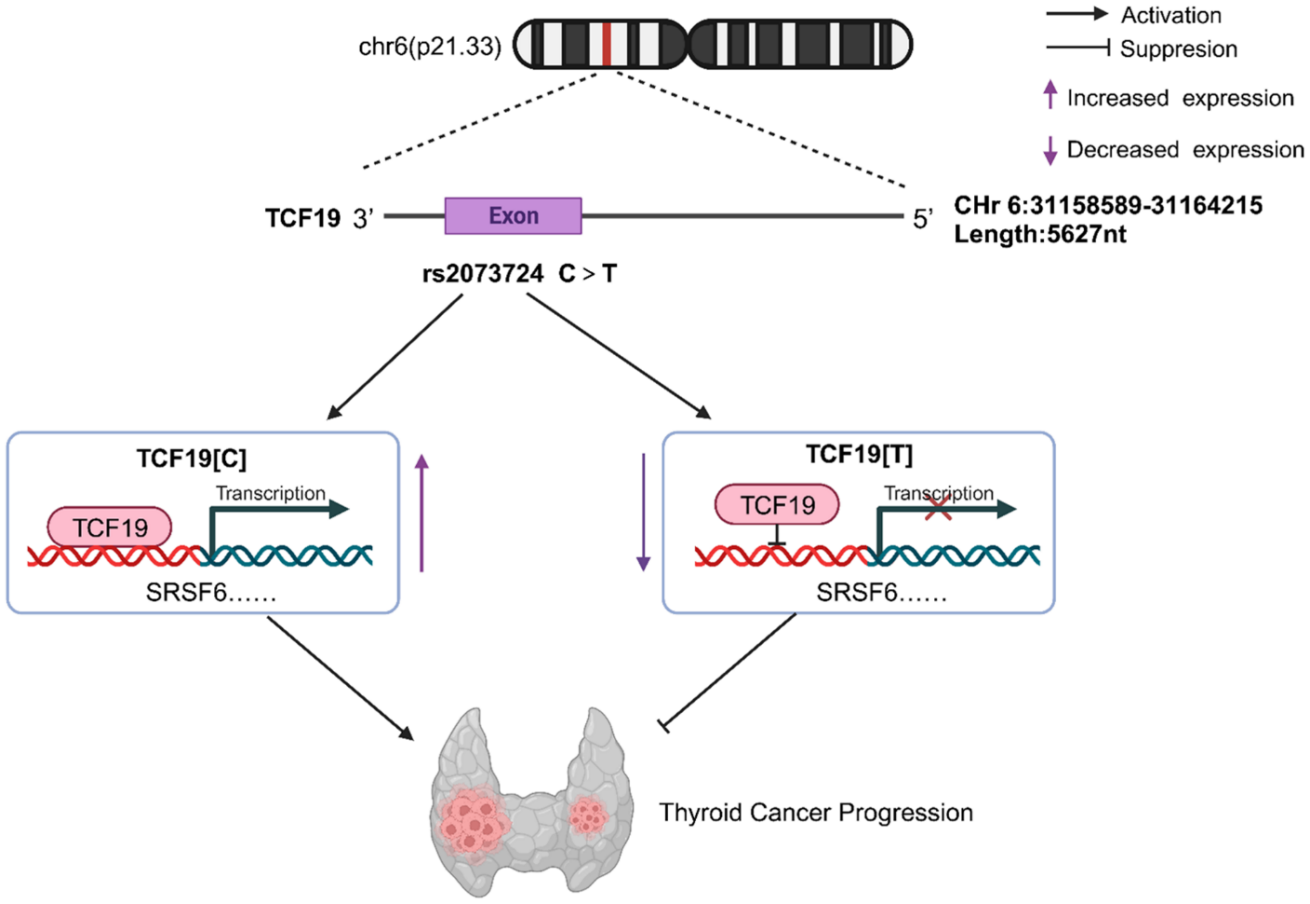
**TCF19 on chromosome 6p21.33 can promote tumor cell proliferation, invasive and migratory ability by enhancing the transcription of SRSF6, etc., thus acting as a tumor-promoting factor in thyroid cancer, while Rs2073724 located in exon 3 of TCF19 negatively regulates the transcription and acts as a tumor suppressor.** The C>T variant of rs2073724 affects the TCF19 function by damaging its transactivating ability.

TCF19 contains a proline-rich region, a forkhead association (FHA) domain, and a PHD finger region [20], indicating that TCF19 is important transcription factor of transcriptional regulation. The FHA domain serves as a nuclear signalling domain or as a phosphoprotein binding domain [21]. Many proteins containing an FHA domain are found in the nucleus and are involved in DNA repair, cell cycle arrest, or pre-mRNA processing [22]. The PHD finger is located at the carboxyl terminus, which may allow it to interact with chromatin via methylated histone H3 [23, 24]. A recent study showed that the PHD finger of TCF19 is necessary for regulating hepatocellular carcinoma cell proliferation through its H3K4me3 binding ability [25]. These characteristics support that TCF19 plays an important role in promoting thyroid cancer cell proliferation as well as other human cancers. Additionally, TCF19 also contains a proline-rich region, a common characteristic of transactivating factors [20]. Importantly, we found that the missense variation (rs2073724) in our study is located in the proline-rich region of the TCF19 protein. Therefore, the TCF19 SNP may affect protein function by damaging its transactivating ability. To confirm this hypothesis, we conducted ChIP-PCR analysis, and the results demonstrated that TCF19[C] but not TCF19[T] could strongly bind to the promoter of the SRSF6 gene. Accumulated evidence has shown that SRSF6 is a potential oncogenic gene that promotes oncogenic splicing in many kinds of cancers [26, 27]. Together, our data suggest that the TCF19 SNP cannot transcriptionally activate oncogenes efficiently, thus playing a protective role in thyroid cancer progression (Figure 5).

A growing body of evidence suggests that TCF19 plays an important role in many types of cancer, including colorectal cancer, non-small cell lung cancer, renal clear cell carcinoma, hepatocellular carcinoma, and head and neck cancer [25, 28–31]. Studies report that TCF19 could promote cell proliferation and contribute to the malignant progression of cancer through mechanisms such as regulating WWC1, inhibiting FOXO1, or activating the ATK/FOXO1 signalling pathway [28, 29, 31]. Moreover, genome-wide association studies (GWAS) have indicated that TCF19 increases the risk of certain types of cancer and multiple autoimmune diseases [32–35]. However, the underlying role of TCF19 in the pathology of thyroiditis and thyroid cancer has not been fully investigated. Our study shows that TCF19 regulates many inflammatory pathways in thyroid cancer, such as the TNF signalling pathway and IL-17 signalling pathway. TCGA analysis further demonstrated that TCF19 overexpression affects immune cell infiltration to promote thyroid cancer immune escape and ICB therapy resistance. TCF19 is a transcription factor involved in the regulation of multiple cellular signalling pathways. There is no direct evidence that TCF19 can promote immune escape. Therefore, more studies are needed to determine the specific role and mechanism of TCF19 in tumour immune escape.

Hashimoto’s thyroiditis (HT) is the most common autoimmune inflammatory pathology of the thyroid and is the main cause of autoimmune hypothyroidism. The cooccurrence of Hashimoto’s thyroiditis and papillary thyroid cancer is prevalent (8–36.4%) and increasing [36]. Thus, the association between thyroiditis and thyroid cancer remains an active focus of research and controversy. Both our study and other studies showed that coexistent HT has been reported to be significantly associated with less aggressive clinicopathologic characteristics of the thyroid [37–39]. In addition, one recent study highlights that thyroiditis plays a protective role in thyroid cancer progression and significantly reduces cancer-related mortality [40]. However, the important molecules that drive the development of inflammation are not particularly well understood. Our study finds that a potential deleterious missense variation (rs2073724) of TCF19 is associated with thyroiditis, and further functional studies demonstrate that this SNP plays a protective role in thyroid cancer development. Moreover, our RNA-seq data also suggest that compared with TCF19[C], TCF19[T] significantly increases the expression of proinflammatory cytokines, such as CCL2, CCL16 and CXCL1. Taken together, TCF19 SNPs could induce a strong inflammatory response in thyroid cancer patients with coexistent HT, thus playing a protective role in thyroid cancer progression.

Our study has several limitations. First, our meta-analysis is based on previously published data, and the quality of the data and potential confounding factors cannot be controlled. Second, we did not identify the TCF19 SNP as a protective factor in thyroid cancer patients with coexistent HT in the meta-analysis of GWAS and pheWAS data. There could be a few reasons: (1) The sample size was too small to detect a significant association. GWAS and pheWAS studies require large sample sizes to have enough power to detect genetic associations, especially for complex diseases such as cancer. (2) The effect size is small. It is possible that the TCF19 variant does confer some protective effect against thyroid cancer in the context of HT, but the magnitude of the effect is small. Even larger sample sizes are required to detect small effect sizes. (3) The TCF19 association is restricted to a specific subgroup. The effect of the variant could be limited to a certain subgroup of thyroid cancer/HT patients based on ethnicity, age, sex, environmental factors, etc. Therefore, the small sample size, small effect size, and subgroup-specific effects could explain the lack of significant TCF19 findings in the current study. In addition, we used an exogenous overexpression system to study the function of TCF19 SNPs in thyroid cancer progression. A CRISPR-Cas9 system to knock-in TCF19[T] in TCF19[C] thyroid cancer cells will lead to more solid results. Finally, we also need transgenic animal models to demonstrate that TCF19[T] inhibits thyroid cancer progression and promotes inflammatory responses in thyroid cancer.

Our study first highlights that TCF19 and missense variation (rs2073724) of TCF19 are potential prognostic biomarkers for thyroid cancer patients. These findings may have important implications for the development of new therapeutic strategies and biomarkers for thyroid cancer and the identification of patients at higher risk for poor outcomes.

## MATERIALS AND METHODS

### Bioinformatics analysis

Previous GWAS and pheWAS studies were collected from multiple sources, including the GWAS catalogue, Opentargets and ExPheWas. The GTEx’s final dataset (V8) contains DNA data from 838 postmortem donors and 17,382 RNA-seq across 54 tissue sites and two cell lines where eQTLs have been well identified and used in previous research. Cancer samples were collected from the TCGA project (N = 10,490). The gene expression level was log2 transformed before the meta-analysis. A fixed effect model and a random effect model were both applied for the aggregation. The 95% CI was applied to show the risk and protective effect on overall survival time. To show more details for different studies, any standardized mean difference (SMD) higher than 3 and lower than -3 is shown with an arrow. Blue filled parallelograms represent the SMD for the fixed effect model and random effect model.

### Cell lines and cell culture

KTC-1 and C643 cells were identified by STR analysis. All cell lines were cultured in RPMI-1640 or DMEM (Gibco, USA) supplemented with 10% foetal bovine serum, penicillin (Gibco, USA) and streptomycin (Gibco) and kept in a humid atmosphere of 5% CO_2_ at 37° C.

### Plasmid construction and lentivirus infection

The cDNA of the TCF19 CDS region was amplified and cloned and inserted into the lentiviral vector pCD-Puro-3×Flag. Catalytic mutant TCF19 was synthesized by GENEWIZ using the same vector as wild-type TCF19. The shRNA targeting human TCF19 was cloned and inserted into pLV-shRNA-puro-mCherry. All constructed vectors were verified by DNA sequencing. The target sequences were as follows: shTCF19-1, 5’-CCAAGGUACUUUGGUCAAUAA-3’; shTCF19-2, 5’-CCCAGGAAGAAACUCCGUGUA-3’; and shTCF19-3, 5’-ACACUGAUCCUAAACUCCAUA-3’. Plasmids were transfected into 293T cells using PEI, Opti-MEM I reduced serum medium (Gibco, USA) and packaging vectors (PAX8 and PVSVG). Forty-eight hours and 72 hours after transfection, viral supernatants were collected, passed through a 0.45 μm filter, and infected at 50% confluency to target cells.

### RNA extraction and RT-qPCR

Total RNA was isolated using RNA Extraction Solution (Servicebio, Wuhan, China), followed by cDNA synthesis using HiScript III RT SuperMix for qPCR (+g DNA wiper) (Vazyme Biotech, Nanjing, China). RNA expression levels were determined by SYBR qPCR Master Mix (Vazyme Biotech, Nanjing, China) on a Bio-Rad QX100 Droplet Digital PCR System (USA), and the RNA expression of each target was calculated by the 2^-ΔΔCT^ method and normalized to that of β-actin. qPCR was performed as shown in Supplementary Table 1.

### Western blotting

Total protein was extracted with RIPA lysis buffer (Solarbio, Beijing, China) containing protease inhibitors. Proteins were detected using BCA protein detection kit (Biosharp, Beijing, China), then separated by SDS-PAGE and migrated to PVDF membrane (Millipore, USA). The membrane was then incubated with primary antibody against GAPDH (1:5000) (Boster, USA, A00227-1), TCF19 (1:1000) (Abcam, UK, ab230005), and FLAG (1:1000) (Sigma, USA, F1804). Western blot bands were detected using a Sparkjade ECL super (Sparkjade, Shandong, China) and an imaging system (Bio-Rad, USA).

### CCK-8 assay

THCA cells were seeded in 96-well plates at a density of 1 × 10^3^ cells per well and then treated with CCK-8 reagent (APExBIO, USA). The absorbance was measured at 450 nm.

### EdU assay

Cells were cultured in 24-well plates and treated with 1 ml medium containing 1 μl EdU (10 mM). After incubation for 2 hours at 37° C and 5% CO_2_, the cells were fixed with 4% paraformaldehyde for 30 minutes and incubated with 0.5% Triton-X-100 in PBS for 20 minutes. The nuclei were stained with Hoechst 33342 dye. The proliferation rate was calculated according to the manufacturer’s instructions (Bioscience, Shanghai, China). In each group, three regions were randomly selected and imaged with a fluorescence microscope (Leica, Wetzlar, Germany).

### Wound healing assay

Cells were seeded and cultured until a 90% confluent monolayer formed and were then scraped with sterile pipette tips and processed in FBS-free medium as indicated in the text. Three areas were randomly selected under the microscope at 0 h and 48 h, and the distance of the cells that migrated to the scratched area was measured.

### Migration and invasion assays

Migration or invasion assays were performed using 24-well plates immersed in 8.0 μm pore size Transwell filters (Corning, USA) with or without precoated diluted Matrigel (Corning, USA). THCA cells (2×10^4) in serum-free medium were added to the upper chamber, and medium containing 10% foetal bovine serum was added to the lower chamber. After incubation at 37° C for 24 h (migration) or 48 h (invasion), cells were fixed on slides and stained with crystal violet (Solarbio, Beijing, China). Infiltrating cells in 3 random fields were then counted under the microscope.

### RNA-seq data analysis

Total RNA was harvested and isolated from stable TCF19 overexpression, TCF19 mutation and scramble control cells. The library was sequenced on an Illumina NovaSeq 6000. Differentially expressed genes were defined as those with fold change >2 or <0.5, p<0.05 and were subjected to Gene Ontology (GO) and Kyoto Encyclopedia of Genes and Genomes (KEGG) pathway enrichment analysis.

### ChIP-PCR assay

ChIP detection was performed using the SimpleChIP®Plus Enzymatic Chromatin IP Kit (magnetic beads) (CST, USA). For ChIP-qPCR detection, the primers designed for the promoter region of SRSF6 are listed in Supplementary Table 1.

### Statistical analysis

All the data presented are from at least three independent biological experiments and are shown as the mean ± SD. An unpaired two-tailed Student’s t test was used for statistical analysis of the experiments. P<0.05 was considered to indicate statistical significance. All data were statistically analysed with GraphPad Prism 7.0.

## Supplementary Material

Supplementary Figures

Supplementary Table 1
