# Supplementary material for: Multidimensional data analysis revealed thyroiditis-associated TCF19 SNP rs2073724 as a highly ranked protective variant in thyroid cancer
Source: Aging (Albany NY). 2024 Apr 4;16(7):6488–509. doi: 10.18632/aging.205718 (PMC11042956; doi:10.18632/aging.205718)
Supplement: Supplementary Figures [file aging-16-205718-s001.pdf]

SUPPLEMENTARY FIGURES

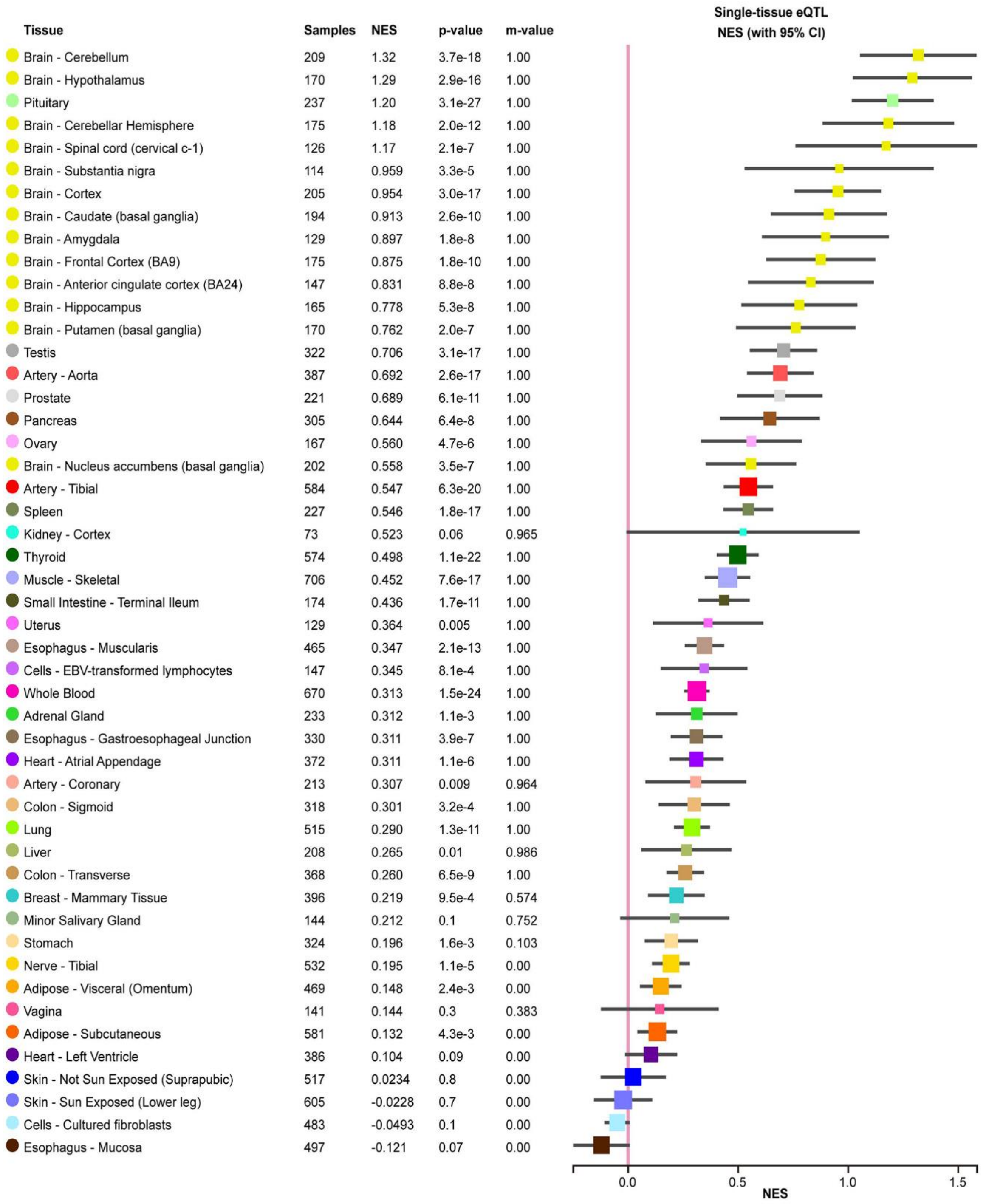

Supplementary Figure 1. Positive expression regulation to TCF19 caused by rs2073724 with GTEx dataset. The GTEx's final dataset (V8) contains DNA data from 838 postmortem donors and 17,382 RNA-seq across 54 tissue sites and two cell lines. In this project, eQTL have been identified with genomics and expression data. GTEx dataset shows rs2073724 alternative allele (Leu) will increase the expression level of TCF19 compared with wild type (Pro) in almost all human tissues (thyroid tissue, NES=0.99, $P=9.6x10^{-66}$) the causal relationship between alterative allele with increased etiology of thyroiditis and thyroid cancer.

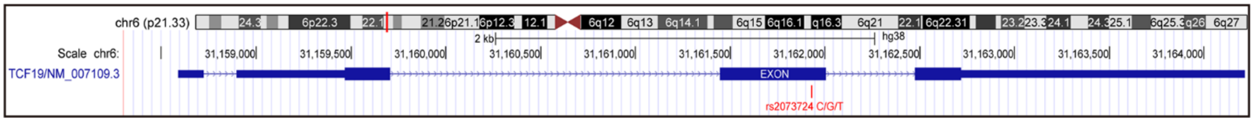

Supplementary Figure 2. RS1317082 is located in the third exon of TCF19 in UCSC.

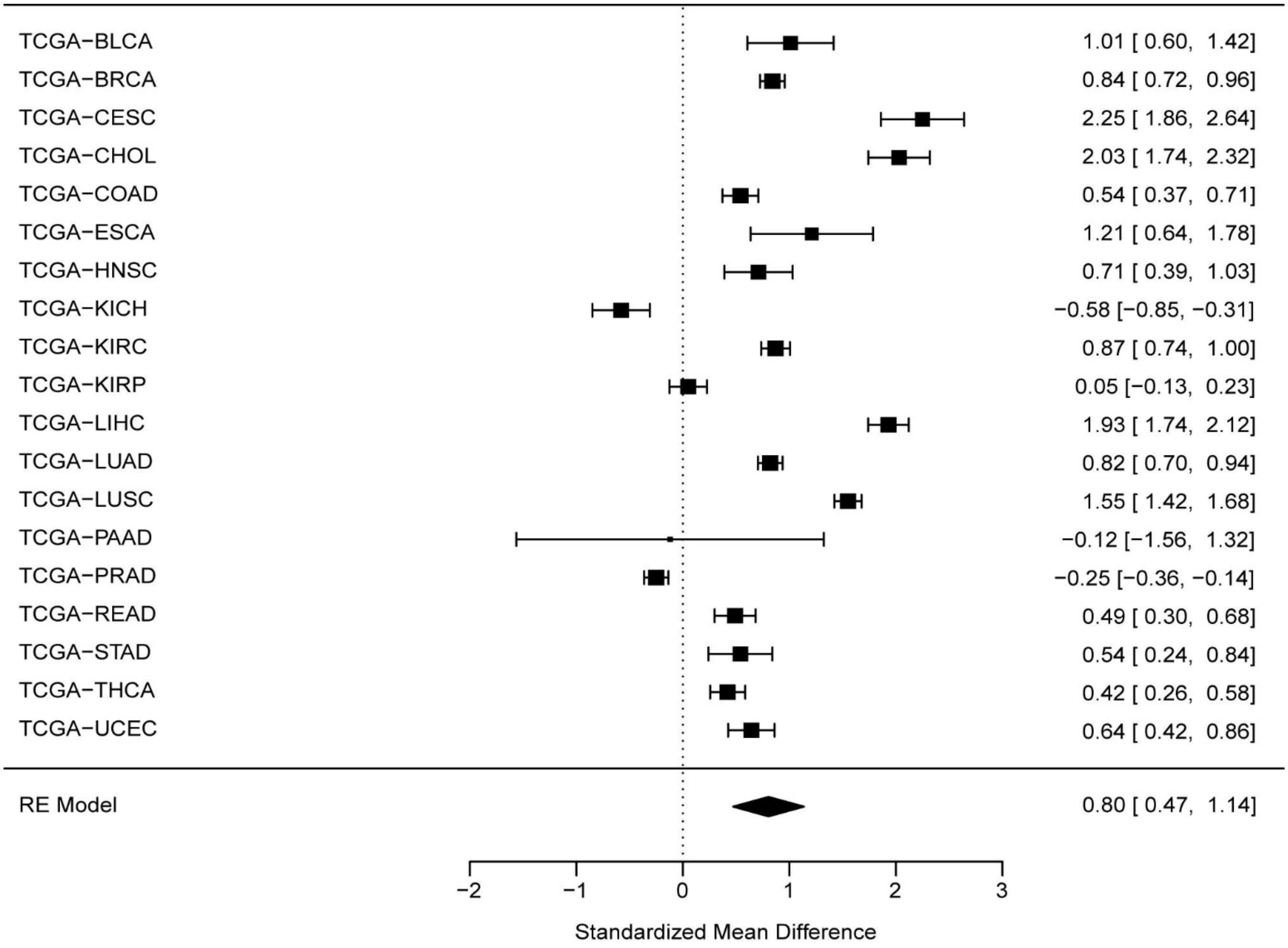

Supplementary Figure 3. Over-expression of TCF19 in human cancers showed with TCGA dataset. Pan-cancer down-regulation of TCF19 crosses 23 types of human cancers. Cancer samples were collected from the TCGA project (N = 10,490). Gene expression level was log2 transformed before the meta-analysis. Both fixed effect model and random effect model were applied for the aggregation. 95% CI was applied to show the risk and protective effect to overall survival time. In order to show more details for different studies, any standardized mean difference (SMD) higher than 3 and lower than -3 was showed with arrow. Blue filled parallelograms represent the SMD for fixed effect model and random effect model.

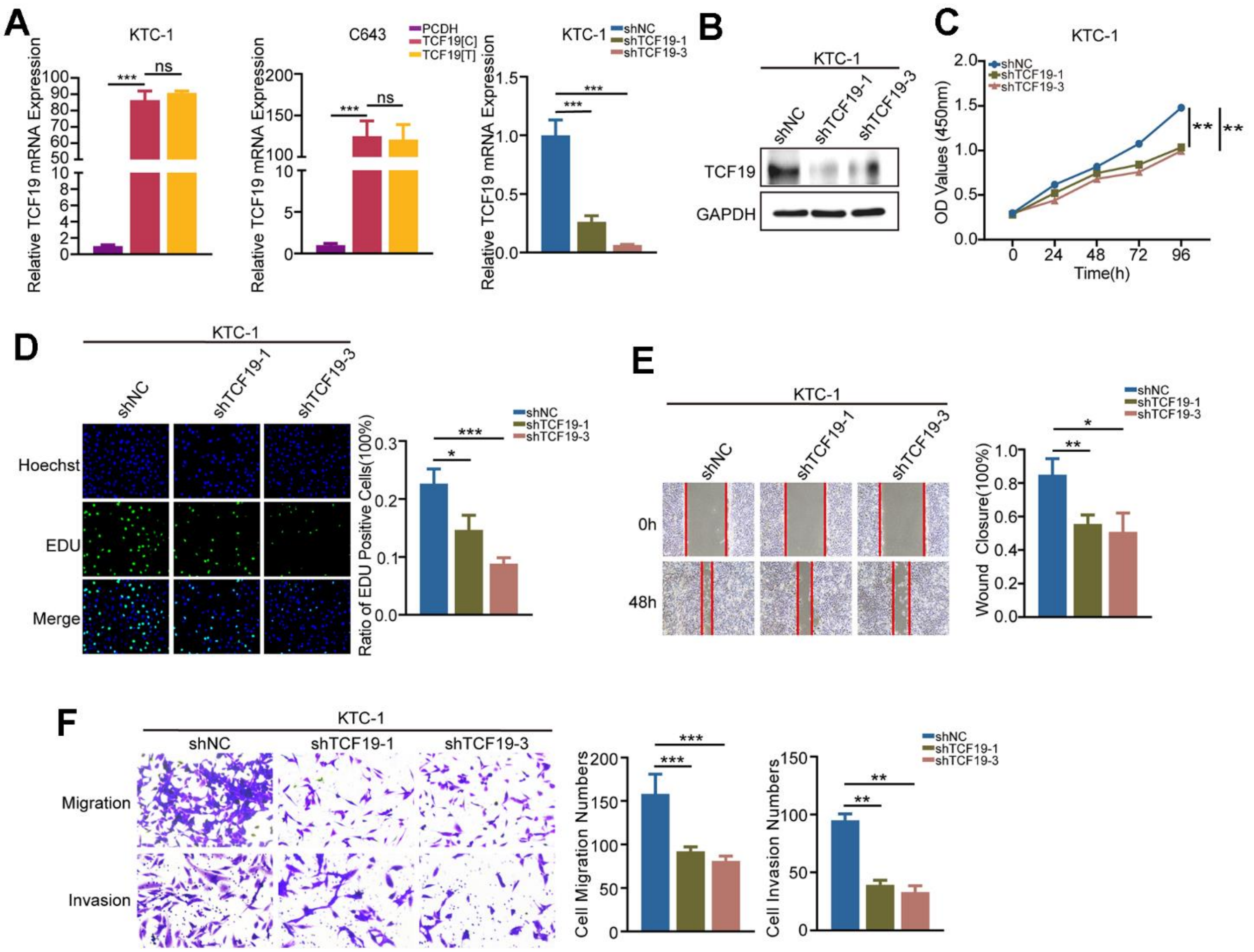

Supplementary Figure 4. Silencing of TCF19 inhibits the proliferation, migration and invasion of thyroid cancer cells. (**A**) TCF19 mRNA expression was detected in KTC-1 and C643 cells transfected with pcDNA, TCF19[C] and TCF19[T] plasmids by qRT-PCR. (**B**) Western blot was used to detect the protein expression of TCF19 in KTC-1 cells stably transfected with PCDH and shTCF19 plasmids. (**C**) CCK8 assay was used to detect cell viability, (**D**) The EdU method was used to detect DNA synthesis. (**E**) Wound healing assay was used to detect cell migration. (**F**) Transwell assay was used to detect cell invasion and migration in KTC-1 cells stably transfected with PCDH and shTCF19 plasmids. Data are shown as means ± S.D. *p<0.05, **p < 0.01, ***p<0.001, NS, not significant.

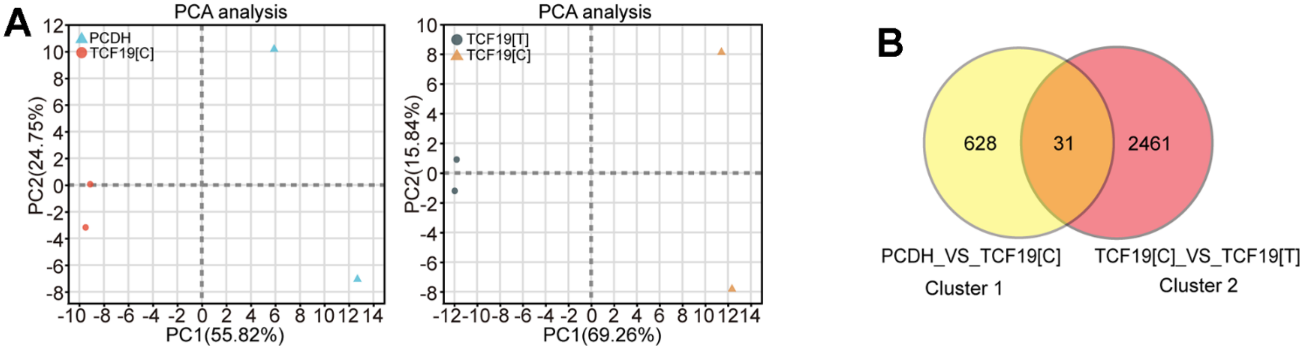

Supplementary Figure 5. Multivariate statistical analysis of RNA-seq samples from different origins. (**A**) PCA score plot based on RNA-seq data. (**B**) Microarray data revealed overlapping cluster 1 (PCDH vs TCF19[C]) and cluster 2 (TCF19[C] VS TCF19[T]) (FC>2.0, P<0.05) in KTC-1 cells, all 31 differential genes listed.

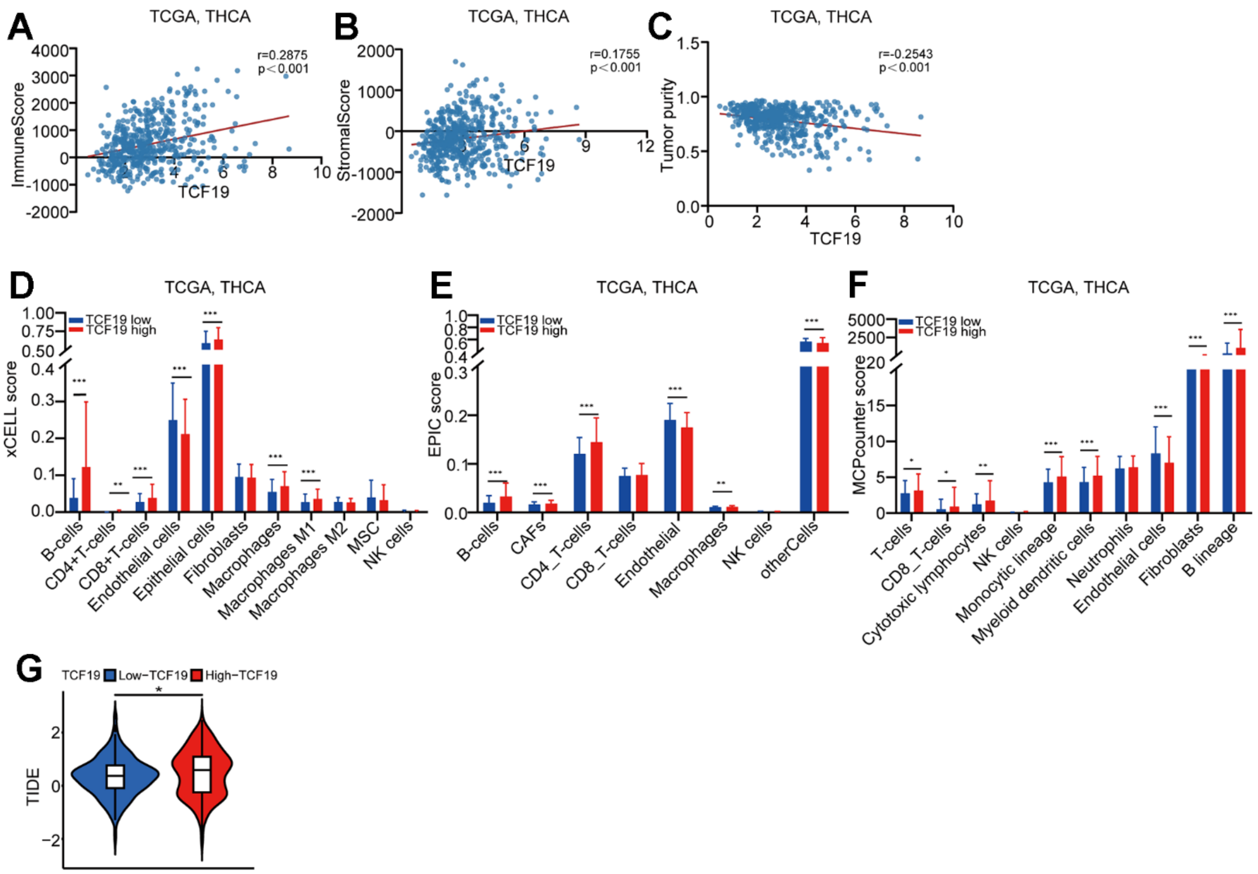

Supplementary Figure 6. TCF19 regulates the TME of thyroid cancer. (**A**–**C**) Correlation analysis of TCF19 expression and three items, including immune cell score, stromal score and tumor purity in THCA tissues from TCGA. (TCGA THCA RNA-seq, n=505, Pearson correlation analysis). (**D**–**G**) XCell, EPIC and MCPcounter analysis of the microenvironmental cells populations and TIDE prediction score for ICB therapy in TCF19 high tissues and TCF19 low tissues. *p*-values were calculated using the Mann-Whitney test.

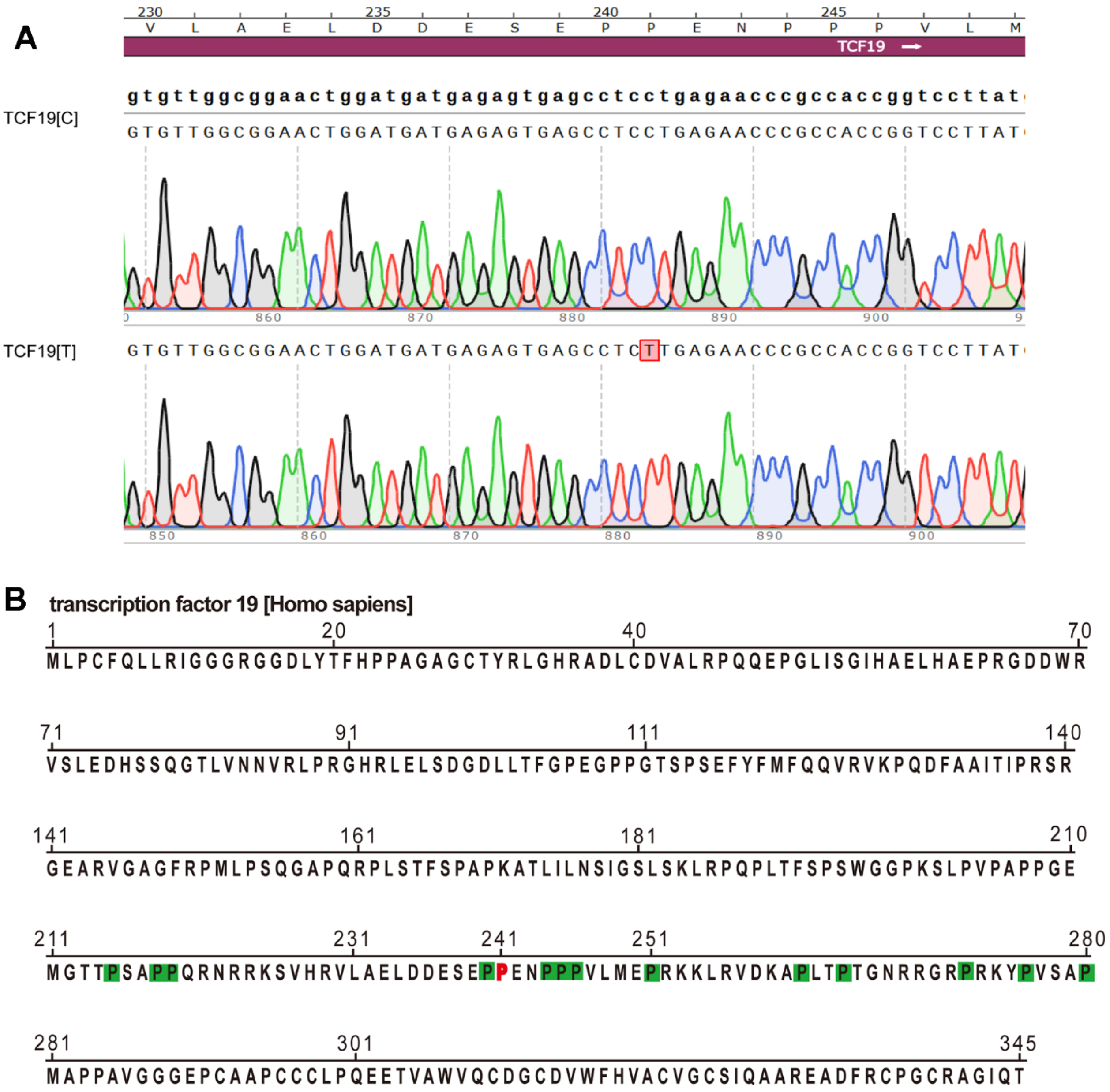

Supplementary Figure 7. TCF19[T] reduces its DNA binding ability, thus affecting protein function. (**A**) Plasmid sequencing peak maps verified TCF19 mutation (P241A) from SnapGene. (**B**) The amino acid sequence of TCF19 protein was from NCBI.
