# Supplementary material for: Multidimensional data analysis revealed thyroiditis-associated TCF19 SNP rs2073724 as a highly ranked protective variant in thyroid cancer
Source: Aging (Albany NY). 2024 Apr 4;16(7):6488–509. doi: 10.18632/aging.205718 (PMC11042956; doi:10.18632/aging.205718)
Supplement: Supplementary Table 1 [file aging-16-205718-s002.pdf]

SUPPLEMENTARY TABLE

Supplementary Table 1. List of primers.

Gene	Forward primer	Reverse primer
Human β-actin	CACCATTGGCAATGAGCGGTTC	AGGTCTTTGCGGATGTCCACGT
Human TCF19	TCAGCCTGGAAGACCACAGCAG	CCAAAGGTCAGGAGGTCTCCAT
Human CSF3	TCCAGGAGAAGCTGGTGAGTGA	CGCTATGGAGTTGGCTCAAGCA
Human SRSF6	GTGCTTTGGACAAACTGGATGGC	CTCCTACTTCGTGACCGTCTTC
Human NCOA5	AGATTCACCGCTCCTGCACAGT	CTGTCTGGCAATCTCCTCACGT
Human IL1B	CCACAGACCTTCCAGGAGAATG	GTGCAGTTCAGTGATCGTACAGG
Human NFE2	GGAGAGATGGAACTGACTTGGC	GAATCTGGGTGGATTGAGCAGG
Human MAT2A	CTGGCAGAACTACGCCGTAATG	GTGTGGACTCTGATGGGAAGCA
Human TXNIP	CAGCAGTGCAAACAGACTTCGG	CTGAGGAAGCTCAAAGCCGAAC
Human GALNT3	CACCTGCAATACTGCTGAAGG	ACAGAGGTTCTAGCCAACCAT
Human SRSF6 Primer 1	GGTTCCTTCCTCCATCTTCAAAGC	AGAGCCACAGTCAGAGAAGCAG
Human SRSF6 Primer 2	GCGGTCCAGCCAGCAGTG	GGGTGACAAAGCAAGACTCCAAC
Human SRSF6 Primer 3	GATAGCCCAATCCACAAGTGAGTC	CCGCCGACATCAGCCTCTC
Human SRSF6 Primer 4	CTGCGGCTGGATTAGAGACATTG	CCCTGGGCTAGATGGTGACTG
Human SRSF6 Primer 5	GCCGCCTCCTCGAAGTAGAC	CCGCTTCTTTCCTTGGAGAGTTC
